# Assembling p53 Activating Peptide With CeO_2_ Nanoparticle to Construct a Metallo-Organic Supermolecule Toward the Synergistic Ferroptosis of Tumor

**DOI:** 10.3389/fbioe.2022.929536

**Published:** 2022-06-28

**Authors:** Jingmei Wang, Wenguang Yang, Xinyuan He, Zhang Zhang, Xiaoqiang Zheng

**Affiliations:** ^1^ Institute for Stem Cell & Regenerative Medicine, The Second Affiliated Hospital of Xi’an Jiaotong University, Xi’an, China; ^2^ Department of Medical Oncology, The First Affiliated Hospital of Xi’an Jiaotong University, Xi’an, China; ^3^ Department of Talent Highland, The First Affiliated Hospital of Xi’an Jiao Tong University, Xi’an, China; ^4^ Department of Infectious Diseases, The Second Affiliated Hospital of Xi’an Jiaotong University, Xi’an, China; ^5^ General Surgery Department, Tang Du Hospital, The Fourth Military Medical University, Xi’an, China

**Keywords:** peptide, p53, supramolecular, protein–protein interactions, anticancer therapy

## Abstract

Inducing lipid peroxidation and subsequent ferroptosis in cancer cells provides a potential approach for anticancer therapy. However, the clinical translation of such therapeutic agents is often hampered by ferroptosis resistance and acquired drug tolerance in host cells. Emerging nanoplatform-based cascade engineering and ferroptosis sensitization by p53 provides a viable rescue strategy. Herein, a metallo-organic supramolecular (Nano-PMI@CeO_2_) toward p53 restoration and subsequent synergistic ferroptosis is constructed, in which the radical generating module-CeO_2_ nanoparticles act as the core, and p53-activator peptide (PMI)-gold precursor polymer is *in situ* reduced and assembled on the CeO_2_ surface as the shell. As expected, Nano-PMI@CeO_2_ effectively reactivated the p53 signaling pathway *in vitro* and *in vivo*, thereby downregulating its downstream gene GPX4. As a result, Nano-PMI@CeO_2_ significantly inhibited tumor progression in the lung cancer allograft model through p53 restoration and sensitized ferroptosis, while maintaining favorable biosafety. Collectively, this work develops a tumor therapeutic with dual functions of inducing ferroptosis and activating p53, demonstrating a potentially viable therapeutic paradigm for sensitizing ferroptosis *via* p53 activation. It also suggests that metallo-organic supramolecule holds great promise in transforming nanomedicine and treating human diseases.

## 1 Introduction

Lung cancer is the largest contributor to tumor-related death around the world. According to statistics, in 2020, the probable number of new cases was 2,206,771, while mortality was 1,796,144 from 185 countries or territories across the world (Bade and Dela Cruz, 2020; Siegel et al., 2021; Sung et al., 2021). Currently, clinical tumor therapeutic options are unsatisfactory. Conventional pharmacotherapies by chemotherapies and/or targeted drugs are often accompanied by cancer recurrence and poor prognosis due to their inherent limitations and complex heterogeneity of cancer. While the emerging immunotherapy revolutionized the medication of lung cancer, it suffers from its intrinsic weakness including a narrow anticancer spectrum, low response rate, and potential toxicity triggered by self-immunity (Crunkhorn, 2020; Kennedy and Salama, 2020). Therefore, innovative precision medicine solutions are urgently needed. To induce other forms of non-apoptotic cell death, such as ferroptosis, overcoming drug resistance points a new direction for cancer therapy.

Ferroptosis, a style of cell death with iron-reliance caused by intracellular lipid peroxidation, has different death characteristics compared to apoptosis, pyroptosis, and autophagy (Jiang et al., 2021). Modulating cellular energy metabolism can significantly affect cellular sensitivity to ferroptosis, given that it is dependent on lipid metabolism and oxidative stress (Conrad and Pratt, 2019). Fortunately, metabolic reprogramming also inevitably occurs during carcinogenesis (Chae et al., 2016), making cancer cells highly sensitive to ferroptosis-inducing therapies (Hu et al., 2020). More importantly, induction of the ferroptosis pathway by depleting Xc or GPX4 has been shown effective in killing drug-resistant cancer cells (Chae et al., 2016). The new study also demonstrates the emerging role of ferroptosis in the crosstalk between tumor cells and immune cells (Hu et al., 2020). It indicates that targeting ferroptosis is of great significance for anticancer therapy. However, cancer cells tend to weaken ferroptosis by increasing the expression of antioxidant enzymes (Li et al., 2022a) or upregulating prominin2 (Brown et al., 2021) to promote iron transport. Abundant ferroptosis targets and regulatory networks provide an available resource for ferroptosis sensitization (Luo et al., 2021a). Among them, p53 as a tumor suppressor can enhance the cell sensitivity to ferroptosis in a direct (transcription-dependent inhibition of SLC7A11 expression) and indirect manner (by regulating amino acid metabolism, iron transport, PUFA metabolism, and antioxidant defense) (Ji et al., 2022).

p53 protein, one of the most important tumor suppressor proteins (Levine, 2020; Liu and Gu, 2021), is often abnormally expressed in most human tumors. In wild-type TP53 tumor types, the expression level and transcriptional function of p53 protein are often negatively regulated by MDM2 and the homolog MDMX, resulting in inhibition of its tumor suppressor function (Ivanov et al., 2013; Wade et al., 2013; Meek, 2015; He et al., 2020). Therefore, the p53-MDM2/MDMX protein interaction is a reasonable and broad therapeutic target in TP53 wild-type tumors. Although a large number of small-molecule drugs that activate p53 have been discovered, such as nutlins and imidazole WK23 (Liu et al., 2019), due to the poor targeting and specificity of small molecules, administration of high concentrations and subsequent biological toxicity is inevitable. Peptide drugs with natural advantages such as high affinity and good biosafety are becoming powerful competitive drugs for protein–protein interaction (PPI) modulators (Yan et al., 2022). At present, there have been many explorations and modifications of p53-activator peptides, and considerable therapeutic effects have been achieved at the animal level (Zheng et al., 2021; Yan et al., 2021). However, searching for higher-affinity peptide segments and overcoming their pharmacological barriers (Giribaldi et al., 2021; Gonzalez-Valdivieso et al., 2021) to promote their clinical translation still have a long way to go.

Supramolecular polymers, different from traditional chemistry molecules, are based on non-covalent interactions between molecules, such as metal coordination and hydrogen bonding, and are attracting increasing attention as nano-drugs (Aida et al., 2012; Zhou et al., 2021). However, general supramolecules are often limited by single functional components, inherent limitations, and complex biological environments, resulting in unsatisfactory therapeutic effects. Nano-platform-based cascade engineering has been ingeniously introduced to optimize this cancer therapy (Chen et al., 2020), in which metallo-organic supramolecules have proven to be an effective and thriving strategy (She et al., 2020; Jin et al., 2021; Liu et al., 2022). It relies on metallo-organic coordination interactions, based on rich geometric structures and connections between ligands and nodes (Chong et al., 2020; Ni et al., 2020). Various metal materials such as gold (Bian et al., 2018; He et al., 2019a; Yan et al., 2020a; He et al., 2020; Zheng et al., 2021; Yan et al., 2021), silver (Fehaid and Taniguchi, 2018; Mi et al., 2021), iron (Shen et al., 2018; Chen et al., 2021), rare earth elements (Yan et al., 2015; Zhang et al., 2017; Niu et al., 2018; Yan et al., 2018; He et al., 2019b), etc., and organic modules such as peptides (He et al., 2018a; He et al., 2018b; Yan et al., 2020b), nucleic acids (She et al., 2020; Li et al., 2022b), small molecules (Liang et al., 2021), etc., are selected as basic building blocks for the self-assembly of metallo-organic supramolecules. Abundant combinatorial options offer greater possibilities for generating highly effective cancer defense strategies, which can generate more therapeutic species or achieve stronger antitumor effects. Although many successful examples of metallo-organic supramolecules have been reported in tumor imaging (Li et al., 2020; Sung et al., 2021), regulation of protein interactions (He et al., 2019a), immunotherapy (Liang et al., 2021; He et al., 2022), and combination therapy (Jin et al., 2021; Liu et al., 2022), great challenges remain in the efficient and simple synthesis of such complex nanosystems.

Herein, to realize the combination of ferroptosis therapy and p53 activation, p53 activator peptide (PMI) and the free-radical generating nanoparticle CeO_2_ were selected to induce ferroptosis in cancer cells (Sugantharaj David et al., 2017). Based on metal-organic coordination and a “one-pot” self-assembly strategy, a bifunctional metal-organic supramolecular (Nano-PMI@CeO_2_) was constructed, in which CeO_2_ functioned as the inorganic building block of supramolecules, while the peptide-gold precursor polymer formed based on gold–sulfur bond functioned as the inorganic building block. The end product, Nano-PMI@CeO_2_ was obtained by the *in situ* reduction and self-assembly based on gold-thiol coordination bonds of peptide-gold precursor on the surface of the CeO_2_ core. Due to the coverage of CeO_2_ by the peptide gold precursor, Nano-PMI@CeO_2_ has good biosafety in normal sites. The reduction of gold–sulfur bonds in the tumor microenvironment triggers the disassembly and release of CeO_2_ and peptides at tumor sites, followed by dual antitumor effects of ferroptosis and p53 activation. In conclusion, this combination therapy is promising to reinvigorate the use of ferroptosis-sensitizing therapy in antitumor therapy.

## 2 Materials and Methods

### 2.1 General Instructions

The synthetical peptides were all purchased from CS bio Co. LLC. The additional chemical reagents in our research were obtained from Sigma-Aldrich, unless otherwise expressly announced.

### 2.2 Synthesis of Nano-PMI@CeO_2_

Under the HBTU/HOBT agreement, the peptides were compounded with an optimized agreement developed for the Fmoc-SPPS methodology, which was based on appropriate resins by the automatic peptide synthesizer (CS Bio 336X). The nanoparticles were prepared through a “two-step, one-pot” gradual chemical reaction under appropriate conditions. In the first step, 2 mg PMI and 2 mg NH_2_-PEG_n_-SH were stirred with 4 ml deionized water, and 1 ml of 10 mM chloroauric acid solution was added at 500 rpm stir for 5 min. During the process, a pale-yellow turbid liquid turned into a purple-red transparent solution in the reaction system, accompanied by an obvious Tyndall effect. In step 2, 5 ml HEPES (100 mM) in which were dissolved 1 mM CeO_2_ nanoparticles, subsequently, was added to the precursor polymer solution for its mild reduction. In addition, to verify the effects of p53 activation and ferroptosis acting independently, forming a univariate experimental control with Nano-PMI@CeO_2_, we substituted PMI-SH with NH_2_-PEG_n_-SH in step 1 to synthesize corresponding nanoparticles termed Nano-PEG@CeO_2_, and Nano-PMI were obtained by replacing CeO_2_ with the prefabricated gold seed solution in step 2, and other conditions remained constant. We also prepared empty carrier gold nanoparticles Nano by replacing PMI-SH and CeO_2_ with NH_2_-PEG-SH and gold seed solution, respectively.

### 2.3 Cell Culture

The A549 cell lines (human) and the Lewis lung carcinoma cells (LLC, mouse) were bought from the Chinese Academy of Science Cell Bank (Shanghai, China), cultured in a standard incubator with the DMEM medium, and supplemented with FBS (10%), penicillin (100 U/ml), and streptomycin (100 μg/ml).

### 2.4 Apoptosis Analysis

Generally, A549 cells were cultured in a 6-well culture dish with a suitable density for 24 h prior to treatments. Then, the cells were incubated with the Nano-PMI@CeO_2_ (0.02 mg/ml), the Nano-PEG@CeO_2_ (0.02 mg/ml), and the Nano for 48 h. Next, all cells were harvested and stained according to the protocol of the FITC PE-7AAD Apoptosis Detection Kit (BD, United States).

### 2.5 Western Blot Analysis

After the indicated treatments of 48 h, the A549 cells were collected and the total protein was extracted. The proteins were separated by polyacrylamide gels after preprocessing, transferred to the nitrocellulose transfer membrane, and probed using primary and then secondary antibodies. The primary antibodies are listed as follows: anti-p53(sc-126, United States), anti-MDM2(sc-13161, United States), anti-GPX4 (sc-166570, United States), anti-SLC7A11 (ab37185, United States), anti-COX2 (12375-1-AP, United States), and anti-GAPDH (60,004-1-lg, United States). The ECL substrate (Millipore, MA, United States) was used for signal visualization. The protein expression of p53, MDM2, GPX4, COX2, and SLC7A11 was normalized to GAPDH and analyzed by ImageJ.

### 2.6 Mouse Study

All C57BL/6 mice were obtained from the Laboratory Animal Center of Xi’an Jiaotong University, providing a standard specific pathogen-free condition. The experimental procedures were approved by The Medical Ethics Committee of Xi’an Jiaotong University.

C57BL/6 mice (aged 5–6 weeks) were age-matched for tumor inoculation. The LLC cell line was inoculated subcutaneously for mice (1 × 10^6^ cells/site). When the volume of the tumor reached ∼100 mm^3^, the mice were selected randomly into the control group, Nano-PMI@CeO_2_ (2 mg/kg), Nano-PMI (2 mg/kg), Nano-PEG@CeO_2_ (2 mg/kg) groups (six mice per group). Treatment was administered *via* intraperitoneal injection once every other day. The body weight and condition of mice were monitored daily. In addition, tumor volumes were analyzed by the following formula: 1/2× major axis ×width-diameter^2^. The humane endpoints were determined based on the level of animal discomfort and tumor sizes.

### 2.7 H&E and Immunohistochemistry

Tissues were stained with hematoxylin–eosin (H&E) referring to regular histopathological techniques. All sections used for histological analysis were 4 μm thick. For immunohistochemistry, primary antibodies were used: anti-p53 (21891-1-AP, United States), anti-COX2 (12375-1-AP, United States), and anti-GPX4 (sc-166570, United States). The slices were scanned with a Scanner, and images were analyzed through ImageJ.

### 2.8 Statistics

Student’s t-test was chosen to test the statistical difference between the experimental results of the two groups of data. ANOVA was used to analyze more intergroup differences, and the Tukey post-analysis or log-rank test was used when necessary (*p < 0.05, **p < 0.01, and ***p < 0.001).

## 3 Results and Discussion

### 3.1 Synthesis and Characterization of Nano-PMI@CeO_2_

**FIGURE 1:**
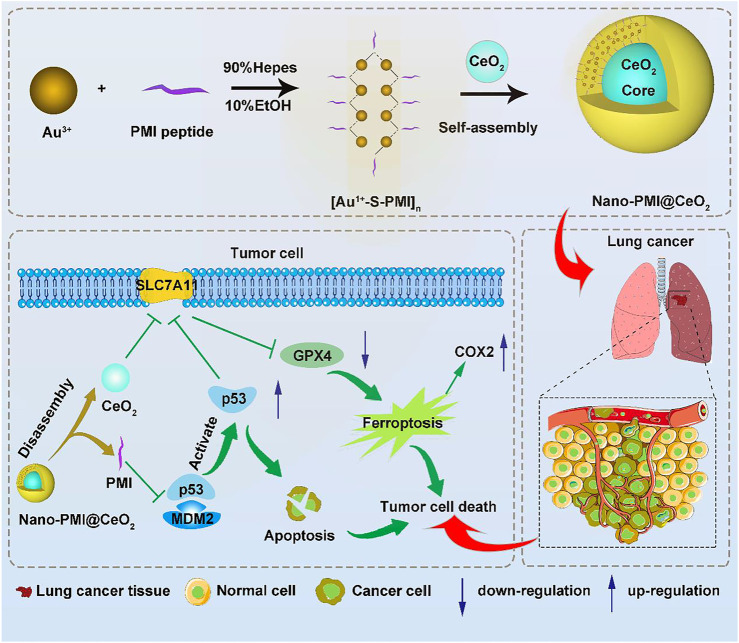
Synthesis and function of Nano-PMI@CeO_2_. Schematic depiction for the synthesis procedure of Nano-PMI@CeO_2_ and their targeting in the lung cancer site by EPR effect and p53 pathways inducing ferroptosis.

**FIGURE 2:**
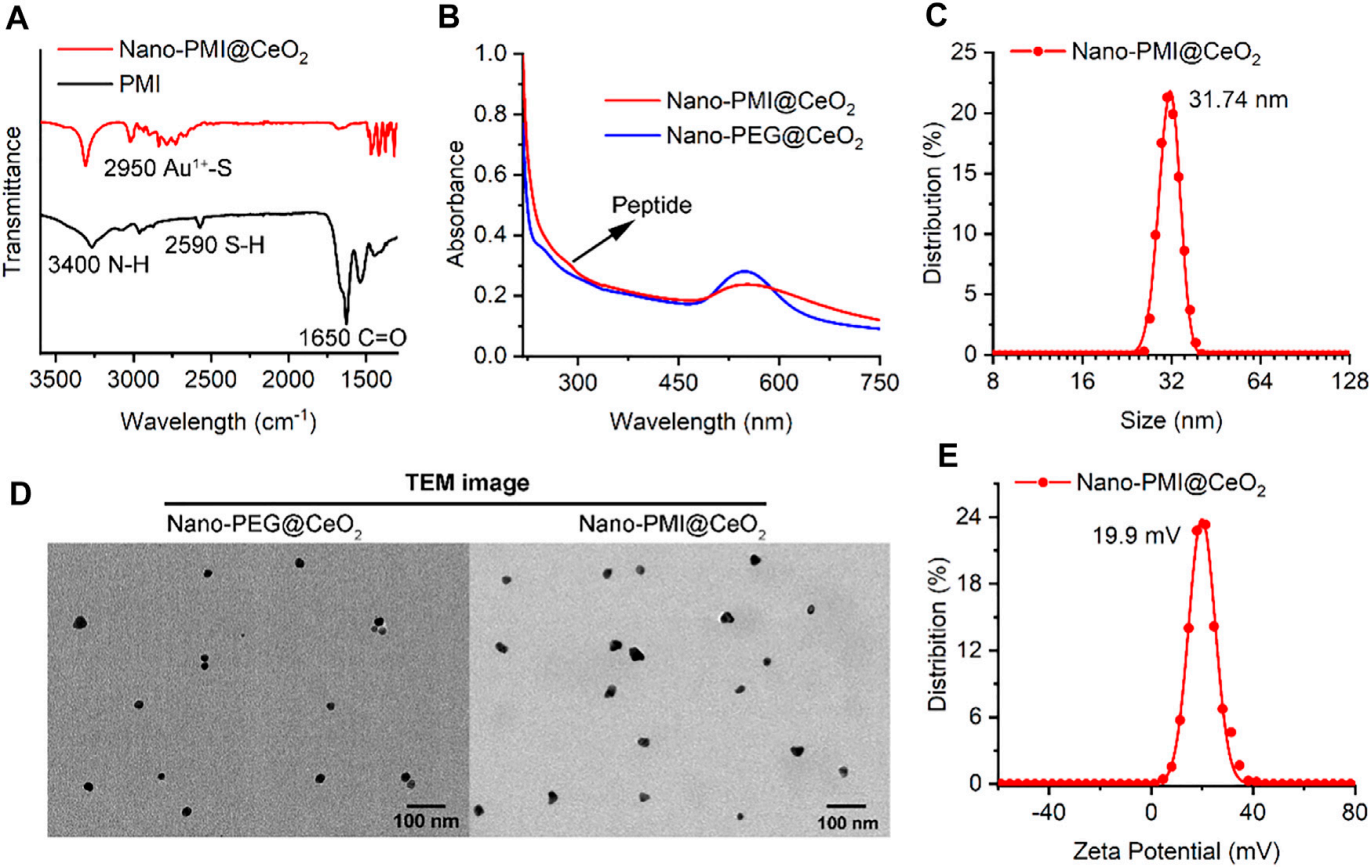
Characterization of Nano-PMI@CeO_2_. **(A)** FT-IR spectroscopy of Nano-PMI@CeO_2_ and PMI. Two absorption peaks at 3400 cm^−1^ and 1650 cm^−1^ were distributed to the stretching vibration of N-H and C=O of peptides. **(B)** UV–Vis spectra of peptides Nano-PMI@CeO_2_ and Nano-PEG@CeO_2_. The typical Au nano shell absorption peak was observed closely at 550 nm. **(C)** Surface charge (Zeta potential) of Nano-PMI@CeO_2_ was measured in PBS at pH 7.4. **(D)** TEM images of Nano-PEG@CeO_2_ and Nano-PMI@CeO_2_. **(E)** Hydrodynamic diameter of the Nano-PMI@CeO_2_ was measured by dynamic light scattering.

To construct this bifunctional metal-organic supramolecular Nano-PMI@CeO_2_ with ferroptosis induction and p53 activation, the choice of basic functional building blocks is crucial. In previous reports (He et al., 2020; Zheng et al., 2021), PMI showed potent regulation of p53-MDM2/MDMX, accompanied with huge nano-engineering work on it, which provided us with great convenience. As for the ferroptosis-inducing module, the rare earth element nanoparticle CeO_2_ was selected. The fabrication of Nano-PMI@CeO_2_ mainly includes two steps: 1) the preparation of PMI peptide-gold precursor polymers [Au^1+^-S-PMI] n and 2) the reduction and self-assembly of peptide-gold precursors on the surface of nanoparticle CeO_2_ (Figure 1). In step 1, the peptide-gold precursor was formed by spontaneous coordination between Au^3+^ in chloroauric acid and thiolated PMI peptides. The disappearance of sulfhydryl groups in PMI-SH and the appearance of Au–S in Nano-PMI@CeO_2_ were confirmed in the Fourier Transform Infrared (FT-IR) spectrum (Figure 2A). PMI could be easily obtained by solid-phase synthesis (SPPS), and the thiolylation of PMI was achieved by introducing a cysteine residue at its C-terminus, which was crucial for the preparation of peptide gold precursor and subsequent self-assembly.

In step 2, 1 mM CeO_2_ nanoparticles were dissolved in 5 ml of 100 mM HEPES and added to precursor polymer solution for its mild reduction. The peptide-gold polymer covered the surface of CeO_2_ and self-assembles under the aurophilic interactions and van der Waals forces. During the process, the reaction system changed from turbid liquid to a purple transparent solution, accompanied by an obvious Tyndall effect. There was no precipitation after the solution was placed at room temperature for 24 h, which indicated the successful preparation of Nano-PMI@CeO_2_ supramolecular colloid. The characteristic absorption peak of the peptide in FT-IR (Figure 2A) and the absorption peak in the UV–Vis absorption spectra (Figure 2B) confirmed that the peptide was integrated. In addition, we also prepared the empty-cargo counterpart of Nano-PMI@CeO_2_, termed Nano-PEG@CeO_2_.

Through dynamic light scattering (DLS), we obtained the particle size of the NPs. The average diameter of Nano-PMI@CeO_2_ was shown to be 31.74 nm (Figure 2C). Under transmission electron microscopy (TEM), both Nano-PMI@CeO_2_ and Nano-PEG@CeO_2_ exhibited good monodisperse properties and uniform size (Figure 2D). The size distribution of Nano-PEG@CeO_2_ and Nano-PMI@CeO_2_ by TEM was in line with the results of DLS (Supplementary Figure S1). Nano-PMI@CeO_2_ had a ζ potential of 19.9 mV in PBS solution (pH = 7.4), which suggested that the nanoparticles had good colloidal stability (Figure 2E). Moreover, the colloidal stability of Nano-PMI@CeO_2_ was proved again by the co-incubation test with 10% FBS, in which Nano-PMI@CeO_2_ maintained its hydrodynamic diameter and ζ potential during the 72 h incubation (Supplementary Figure S2).

To identify the composition of Nano-PMI@CeO_2_, we centrifugally removed the nanoparticle and quantified the residual in the supernatant. First, the nanoparticles were centrifuged at high speed (10,000 g × 10 min), and HPLC was used to detect the polypeptide content in the supernatant. As shown in Supplementary Figure S3, there was almost no residual polypeptide in the supernatant. The nanoparticles were incubated with a high concentration of dithiothreitol (DTT) to disrupt the binding of peptides and gold and passed through the HPLC column again, and the peptide loading in the nanoparticles was calculated to be 91.8%. Furthermore, the Au and Ce elements in the epipelagic liquor were analyzed by inductively coupled plasma mass spectrometry (ICP-MS), and the results (Supplementary Table S1) showed that the Au and Ce elements in the supernatant accounted for 2.2% and 0.4% of the reactants, respectively. Calculated from the ratio of the reactants, the elemental concentrations of gold and cerium in the particles were 0.197 mg/ml and 0.04 mg/ml, respectively, which showed that our nanoparticles contain almost all the gold and Ce elements. Thus, the resulting nanoparticle solution had almost no impurities remaining, and purification is unnecessary. To validate the biodistribution of the Nano-PMI@CeO_2_ *in vivo*, the ^197^Au in the blood, the main organs, and the tumor extracted from LLC-bearing C57BL/6 mice were analyzed *via* ICP-MS. The noticeable blood cycle time of Nano-PMI@CeO_2_ (Supplementary Figure S4) was supported by the metabolic level measured *via* time-based ICP-MS. Nano-PMI@CeO_2_ exhibited low normal tissue storage in a period of 4 h ∼ 1 week due to the metabolism and elimination, while the cumulation of Nano-PMI@CeO_2_ at the tumor focus was high due to the EPR effect. Furthermore, quantitative analysis of Au and Ce elements in the dissociated organs from different time points showed that Nano-PMI@CeO_2_ could be cleared from the body by the mononuclear phagocytosis system. In summary, Nano-PMI@CeO_2_ was co-self-assembly constructed as a metallo-organic supermolecule based on CeO_2_ nanoparticle (metal part) and peptide PMI (organic part), with advantages of stable transport and controlled release for intracellular peptide.

### 3.2 Nano-PMI@CeO_2_ Reactivated p53 Signaling and Augmented Ferroptosis of Lung Cancer *in Vitro*

**FIGURE 3:**
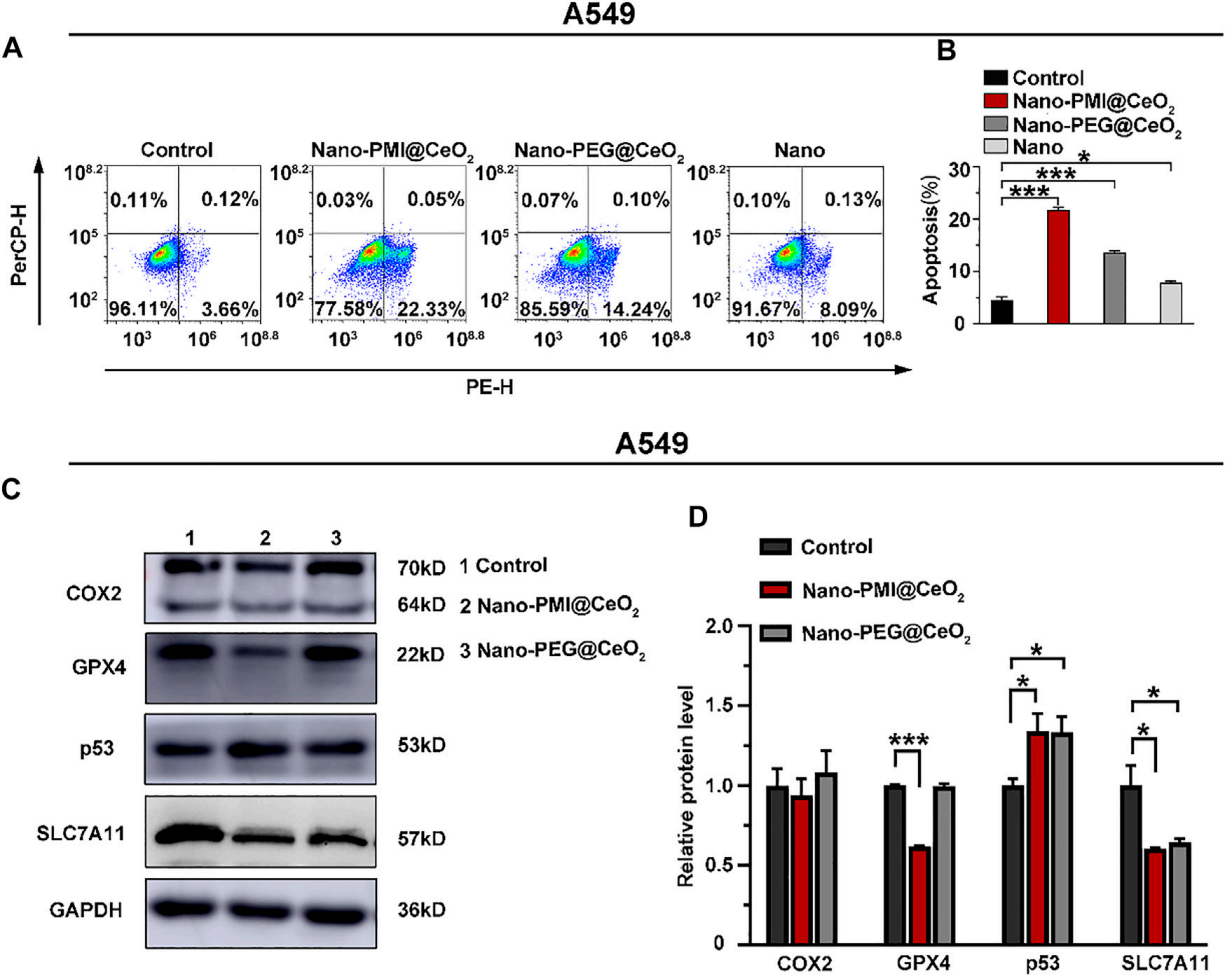
Nano-PMI@CeO_2_ potently enhanced tumor apoptosis *in vitro* by targeting p53 pathways and inducing ferroptosis. **(A)** Apoptosis effects of these NPs on the A549 cell line measured by flow cytometric analysis. **(B)** Apoptosis rate was showed as mean ± SE (*n* = 3). *p* values were calculated by *t*-test (*, *p* < 0.05; **, *p* < 0.01; ***, *p* < 0.001). **(C)** Protein expression was shown of COX2, GPX4, p53, and SLC7A11 in A549 cell line treatment with 0.02 mg/ml Nano-PMI@CeO_2_, 0.02 mg/ml Nano-PEG@CeO_2_ using Western blot. **(D)** Relative protein levels of COX2, GPX4, p53, and SLC7A11 were calculated using ImageJ. Experiment results were presented as mean ± SE (*n* = 3) *p* values were calculated by *t*-test (*, *p* < 0.05; **, *p* < 0.01; ***, *p* < 0.001).

To explore the potential of Nano-PMI@CeO_2_ nanoparticles for suppressing tumor growth *in vitro*, the antitumor mechanism of Nano-PMI@CeO_2_ (0.02 mg/ml) was first tested on the lung cancer cells A549 carrying wild-type p53 and overexpression of MDM2/MDMX. After cells were treated with 0.02 mg/ml Nano-PMI@CeO_2_, Nano-PEG@CeO_2_, and Nano for 48 h, flow cytometric quantification of the increase in the number of PI and Annexin V in different treatment groups was carried out. In contrast to the control group, the apoptotic cell ratio in the Nano-PMI@CeO_2_ group significantly increased by more than 70% (Figures 3A,B), while the Nano-PEG@CeO_2_ group also showed some potent activity in the A549 cells. We designed Nano-PMI@CeO_2_ to induce tumor cell death *via* the ferroptosis and apoptosis hybrid pathway, in which ferroptosis played an important position. Although there was only a minor increase in the Nano-PMI@CeO_2_ group compared to the Nano-PEG@CeO_2_, this evidence still suggested that Nano-PMI@CeO_2_ has potentially suppressed the proliferation process in tumor cells by inducing apoptosis.

Next, we also used the HCT116^−/−^ cells, a p53 knockout cell line, and the NCI-H1975 cells, a p53 mutation cell line, to further verify the cytocompatibility of the Nano-PMI@CeO_2._ Apoptosis experiments showed that after Nano-PMI@CeO_2_ (0.02 mg/ml) treatment for 48 h, there was no significant increase in the apoptosis rate in the p53 knockout cell lines (Supplementary Figures S5A,B), and the p53 mutant cell lines showed almost the same content (Supplementary Figures S5C,D). This result indicated that Nano-PMI@CeO_2_ was dependent on p53 activation to upregulate p53 levels. The apoptosis rate of HUVECs treated with Nano-PMI@CeO_2_ (0.02 mg/ml) for 48 h analyzed by flow cytometry was consistent with the control, which reflected the specific killing effect of the nanoparticles on tumor cells (Supplementary Figures S5E,F).

After demonstrating the Nano-PMI@CeO_2_ exact antitumor effect on the lung cancer cell lines, we further explored the underlying mechanism through Western blotting. Since then, with the treatment indicated with 0.02 mg/ml concentration for 48 h, we harvested the protein of A549 cells. Notably, CeO_2_, a well-known oxidative stress inducer, could trigger the production of OH and iron death in tumors (Ha et al., 2018; Das et al., 2013). As displayed in Figure 3C, the expression of SLC7A11 in the Nano-PEG@CeO_2_ group was significantly downregulated, in comparison with the control group. It was reflected that Nano-PEG@CeO_2_ could regulate ferroptosis of tumor cells, as similarly reported before (Hong et al., 2021; Luo et al., 2021b). Furthermore, the expression of p53 with Nano-PMI@CeO_2_ treatment was remarkably increased compared with the control group. By contrast, the MDM2 was markedly downregulated in the Nano-PMI@CeO_2_ group (Supplementary Figure S6). It was demonstrated that Nano-PMI@CeO_2_ could achieve p53 accumulation in A549 cells by blocking the p53 and MDM2 interactions (Figures 3C,D). In addition, owing to the oxidative stress environment by CeO_2_, reactivating p53 could significantly downregulate the intracellular concentrations of SLC7A11 and GPX4 (Figures 3C,D). These key protein expression levels reflected that Nano-PMI@CeO_2_ was helpful in further augmenting ferroptosis in A549 cells (Jiang et al., 2015; Lei et al., 2021). Taken together, these results demonstrated that Nano-PMI@CeO_2_ not only induced tumor cell deaths by promoting the apoptosis pathway but also owed to augment ferroptosis *in vitro* through reactivation of the p53 pathway.

### 3.3 Nano-PMI@CeO_2_ Suppresses Tumor Progression *In Vivo*

**FIGURE 4:**
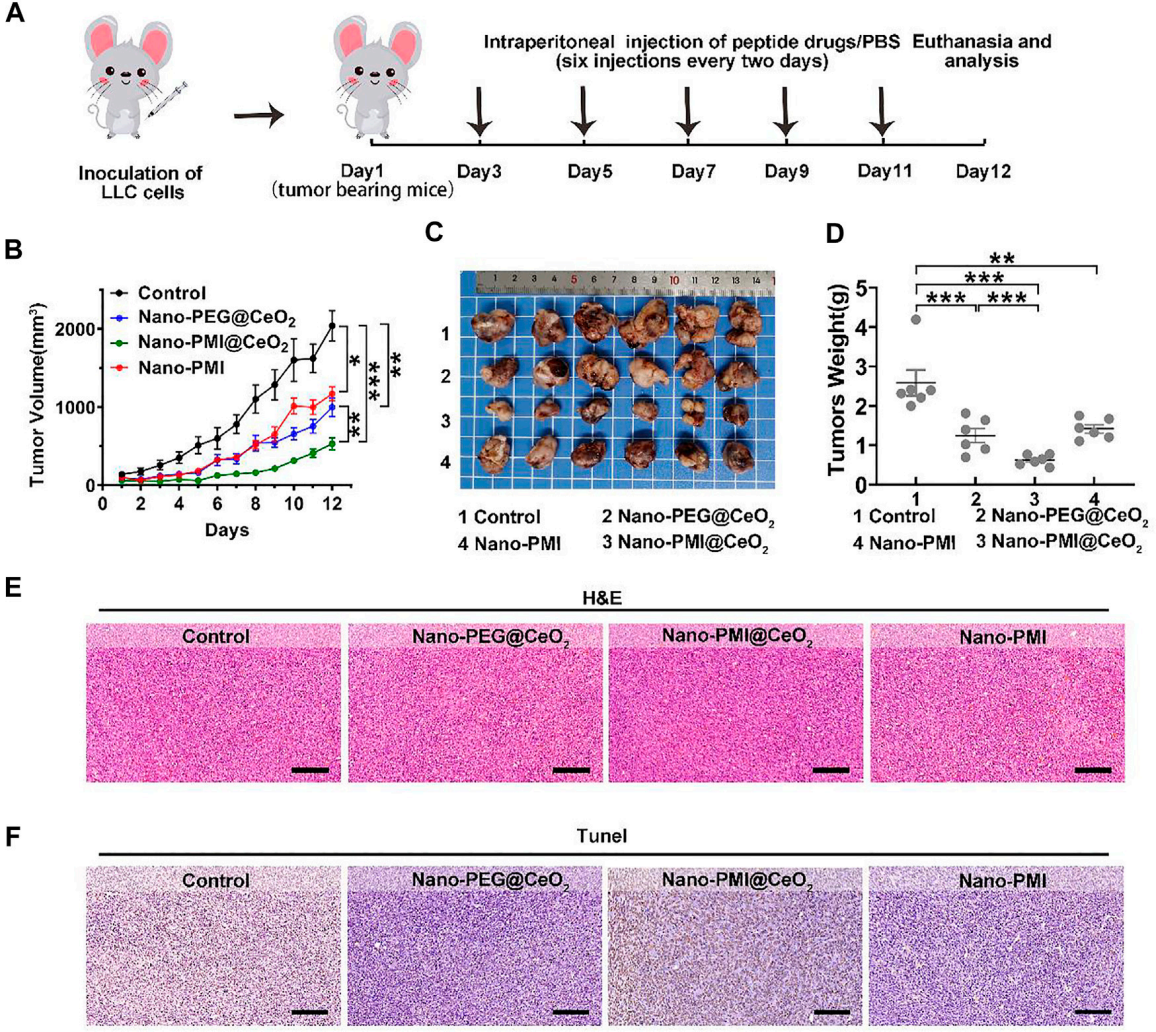
Nano-PMI@CeO_2_ *in vivo* antitumor activity. **(A)** Schematic depiction of the subcutaneous allograft lung cancer model and therapeutic process LLC allograft lung cancer model was established by subcutaneous injection LLC cells. Mice were treated with intraperitoneal injection every 2 days six times with nanoparticle drugs (2 mg/kg) or PBS. **(B)** Tumor sizes were measured by a vernier caliper every day. Tumor volume data were shown as mean ± SE (*n* = 6/group). *p* values were calculated by *t*-test (*, *p* < 0.05; **, *p* < 0.01; ***, *p* < 0.001).**(C)** Photographs and **(D)** average weights of tumors were collected at the end of the experiment (*n* = 6). *p* values were calculated by *t*-test (*, *p* < 0.05; **, *p* < 0.01; ***, *p* < 0.001). (E&F) Allograft tumors from mice after the 12 days of treatment staining by H&E **(E)** and TUNEL **(F)**. (Scale bar: 200 μm).

In the process of further verification of the *in vivo* therapeutic effect of Nano-PMI@CeO_2_, lung cancer allografts were constructed for animal models. In detail, it was achieved by seeding LLC cells (1 × 10^6^/mouse) into the epidermis of C57BL/6, as described in Figure 4A. The Nano-PEG@CeO_2_ (2 mg/kg), Nano-PMI@CeO_2_ (2 mg/kg), and Nano-PMI (2 mg/kg) were injected intraperitoneally every 2 days after the tumor grew to around 100 mm^3^. The tumor volume and body weight were recorded every day. Compared with the group treated with PBS, Nano-PEG@CeO_2_ inhibited tumor proliferation by 51% at the end of treatment (Figure 4B). Also, Nano-PMI@CeO_2_ successfully suppressed the tumor growth with a tumor inhibition rate greater than 74% (Figure 4B), limiting the tumor volume to <550 mm^3^. The comparison of the operated tumors at the end of the 12-day experimental process revealed that tumor growth was noticeably hindered in the Nano-PMI@CeO_2_ treatment group (Figure 4C). At the same time, the tumor weight (Figure 4D) also proved the highest efficiency of Nano-PMI@CeO_2_ compared to the other two groups. In addition, in the survival curve experiment (Supplementary Figure S7), Nano-PMI@CeO_2_ greatly prolonged the median survival time of mice (26 days), significantly surpassing other control groups (19.5 days for Control, 24 days for Nano-PEG@CeO_2_ and 21 days for Nano-PMI). Collectively, these data demonstrated that Nano-PMI@CeO_2_ was a potent antitumor therapy therapeutic agent. In addition, H&E staining assays (Figure 4E) and TUNEL staining assays (Figure 4F) with the quantitative analysis (Supplementary Figure S8) of the tumor tissue further confirmed the superior therapeutic effect of Nano-PMI@CeO_2_. In short, this evidence suggested that Nano-PMI@CeO_2_, as a novel nano-drug, was strongly efficacious in inducing a cancer-killing effect *in vivo*.

### 3.4 Nano-PMI@CeO_2_ Augmented Ferroptosis Through p53 Accumulation *In Vivo*

**FIGURE 5:**
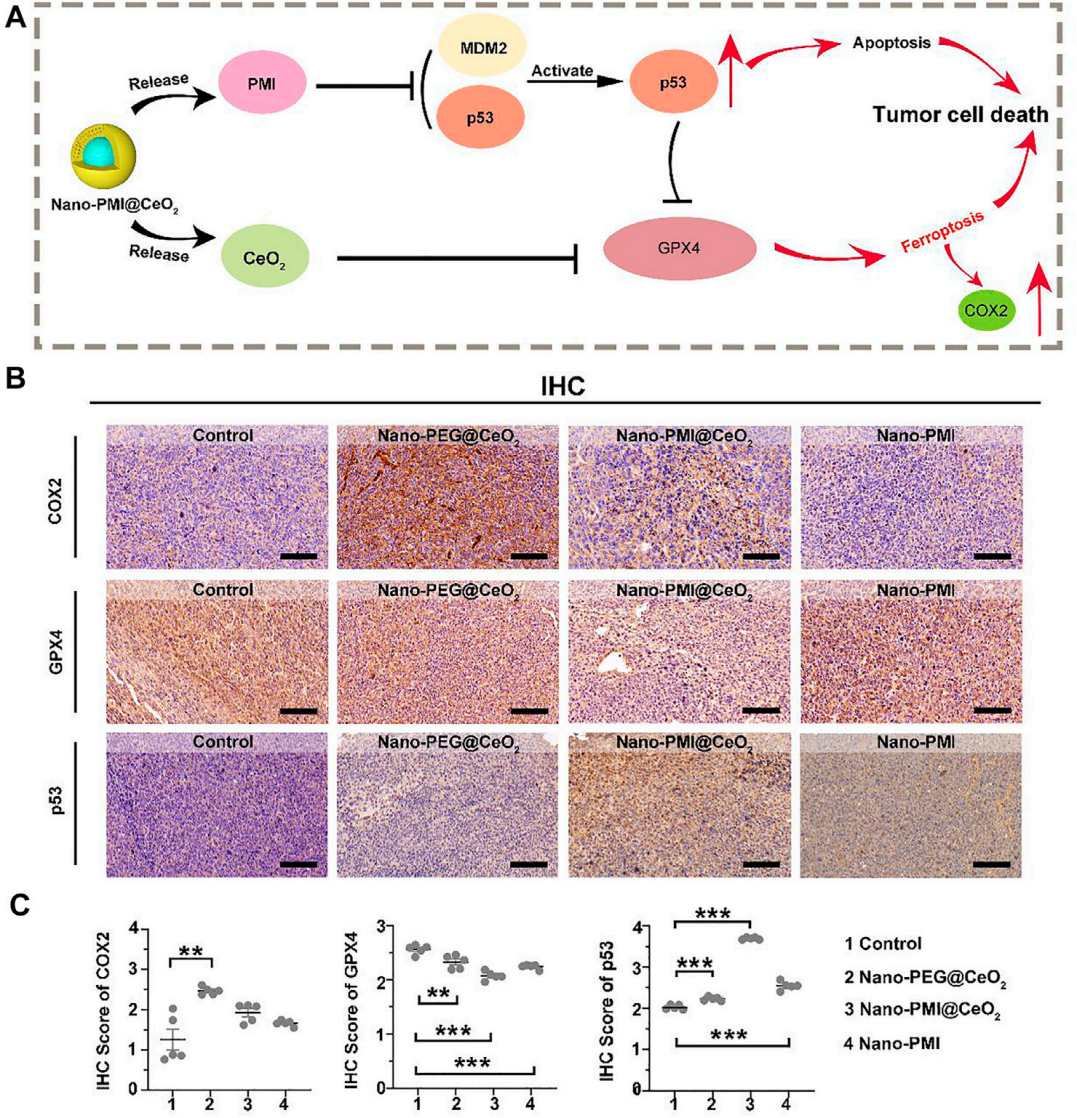
Mechanism of Nano-PMI@CeO_2_ *in vivo* induced tumor cell death. **(A)** Schematic diagram for antitumor activity of Nano-PMI@CeO_2_ targeting the p53 pathways and inducing ferroptosis. **(B)** Representative IHC staining of COX2, GPX4, and p53 in tumor sections. (Scale bar:100 μm). **(C)** IHC scores analysis presented intratumoral protein levels of p53, GPX4, and COX2. *p* values were calculated by *t*-test (*, *p* < 0.05; **, *p* < 0.01; ***, *p* < 0.001).

Based on the results above, we reckoned that Nano-PMI@CeO_2_ could reactivate the p53 pathway by inhibiting the negative regulation of MDM2/MDMX. Under this, the protein levels of SLC7A11 were reduced, and downregulation of GPX4 induced far more ferroptotic cell death when compared to Nano-PEG@CeO_2_ *in vivo* (Figure 5A). To further reveal the underlying mechanisms of Nano-PMI@CeO_2_ on antitumor effect *in vivo*, immunohistochemical staining was used to validate the p53 expression levels and other key proteins relating to ferroptosis. From Figures 5B,C, we could observe that the nanoparticles CeO_2_ inhibited GPX4 protein expression and triggered COX2 protein. Based on this, we reckoned that the nanoparticles CeO_2_ lead to the tumor cell ferroptosis *via* the GPX4 pathway. At the same time, the results showed that the Nano-PMI@CeO_2_ could effectively activate the accumulation of p53 protein in tumor cells *in vivo,* while inducing the ferroptosis process with noticeable downregulation of GPX4 (Figures 5B,C). As a result, Nano-PMI@CeO_2_ earned more active inhibition of tumor proliferation than the Nano-PEG@CeO_2_ group in the regimen. Nano-PMI@CeO_2_ could not only effectively activate the accumulation of p53 protein in tumor cells *in vivo* but also induce the noticeable downregulation of GPX4 (Figure 5). Overall, owing to the enhanced tumor permeability and retention (EPR) effects, the metallo-organic supramolecule could passively be accumulated in the tumor location *in vivo*, validating their potential for facilitating p53 reactivation, while achieving tumor cell apoptosis and enhanced tumor cell ferroptosis.

### 3.5 Nano-PMI@CeO_2_ Safety Evaluation *In Vivo*

**FIGURE 6:**
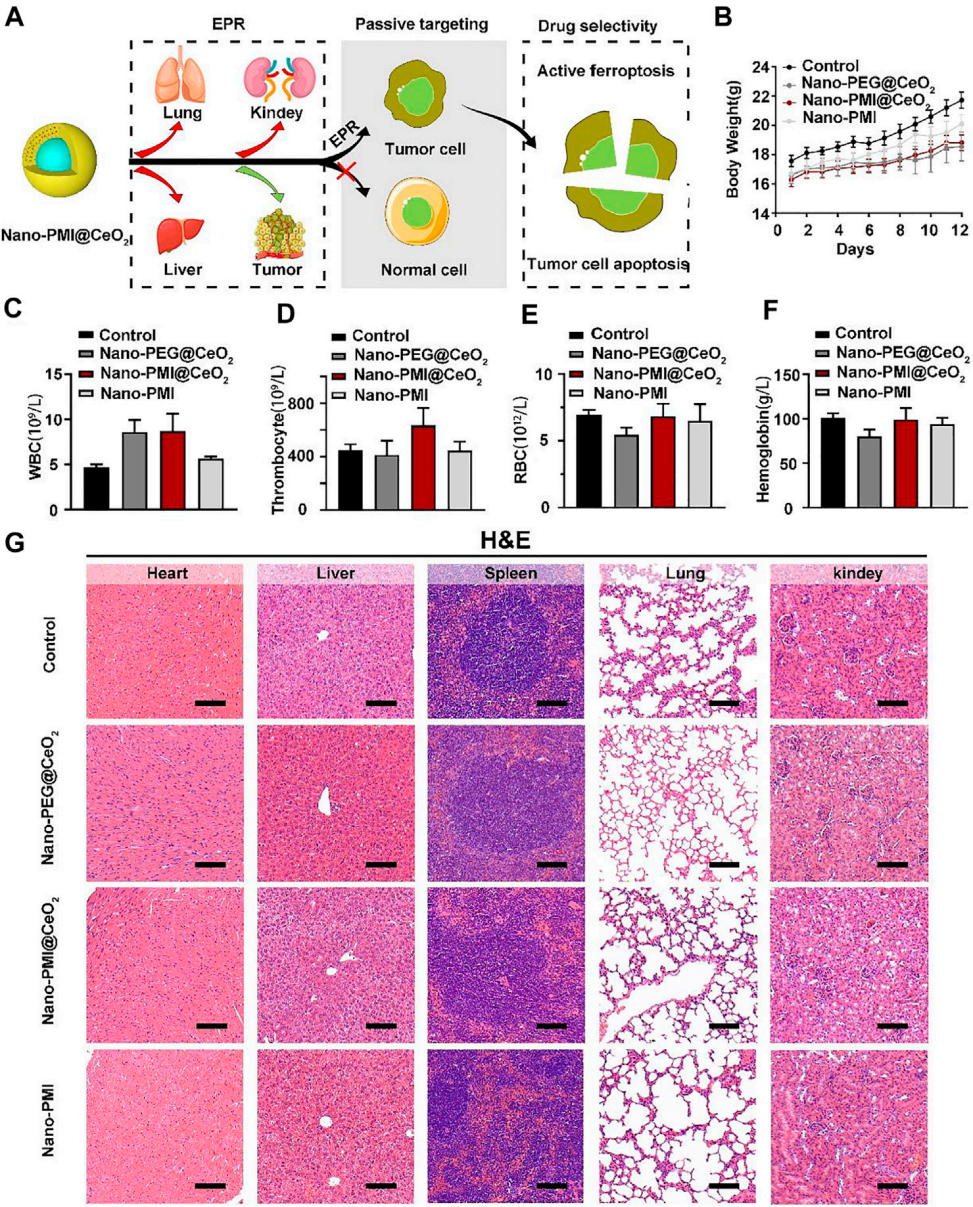
Nano-PMI@CeO_2_ safety evaluation *in vivo*. **(A)** Schematic illustration of tumor specificity for Nano-PMI@CeO_2_ *via* the EPR effect. **(B)** Body weights were measured every day to evaluate the safety of Nano-PMI@CeO_2_, Nano-PEG@CeO_2,_ and Nano-PMI *in vivo*. **(C–F)** Count of white blood cells (WBCs) **(C)**, thrombocytes **(D)**, red blood cells (RBCs) **(E)**, and hemoglobin **(F)** in C57BL/6 mice with the different treatments. **(G)** Representative histological H&E staining images of the heart, liver, spleen, lung, and kidney in mice with the indicated treatment. (scale bar: 100 μm).

Metallo-organic supramolecules often enhance the therapeutic performance *via* reducing functional molecule concentrations in normal tissues and increasing concentrations in tumors sufficiently under the EPR effect (Figure 6A). To evaluate the biosafety with Nano-PMI@CeO_2_ treatment *in vivo*, we performed comprehensive toxicity research using C57BL/6 mice. The Nano-PMI@CeO_2_ was administered intraperitoneally (2 mg/kg) to the mice on alternate days for 12 days. Then, we recorded the changes in body weight of each group, as shown in Figure 6B, and the body weight of mice in the four groups gradually increased. Although the growth rate of body weight in the Nano-PMI@CeO_2_ group was slightly lower than that in the control group and Nano-PMI group at the later stage of treatment, there was no remarkable difference between the groups. Then, as expected, the safety of both Nano-PMI@CeO_2_ and Nano-PEG@CeO_2_ was further confirmed by analysis of white blood cells (Figure 6C), thrombocytes (Figure 6D), red blood cells (RBCs) (Figure 6E), and hemoglobin (Figure 6F) in peripheral blood of mice. The H&E staining for the key organ slice also confirmed the abovementioned results that Nano-PMI@CeO_2_ was enough biosecurity as a potential therapeutic effect (Figure 6G). The meaning of this research was to verify the antitumor and biosafety of Nano-PMI@CeO_2_ for the treatment of lung cancer to identify evidence-based resources that could better facilitate informed consent.

## 4 Conclusion

Ferroptosis, as a novel tumor therapy strategy, has gained a great lot of attention in tumor development and treatment. As the primary hallmark of cancer is a valid escape from conventional modes of cell death, the traditional cancer therapeutic schedules still face enormous challenges, covering drug resistance, off-target effects, and so on (Shan et al., 2020; Luo et al., 2021a). Recently, nanoparticles have provided a new form of opportunity for anticancer therapy because of ferroptosis activation. For example, Zhao et al. developed a micellar delivery nano-drug, called DHM@RSL3, to release RSL3 in the hypoxia environment around the tumor, suppressed GPX4 protein expression with site-selectivity, and induced ferroptosis (Guo et al., 2020). Furthermore, Lin et al. constructed an arginine-capped silicate nano, named AMSNs, which presented huge responsiveness of GSH to activate GPX4-related ferroptosis in tumors (Wang et al., 2018). Beyond that, compared to small molecules, nanomaterials had higher power of clinical application in inducing iron death, taking advancements of longer blood circulation, stronger targeting, more controllable release ability, etc. Therefore, nanoparticle-induced iron death is considered an effective and safe way for various malignant tumor treatments.

Although multiple advances have been tapped out to produce iron death in malignancy tumors, the nano-drug as a single ferroptosis strategy may be unsatisfied with the demands of the complex tumor situation, such as drug resistance (Zhang et al., 2019; Guo et al., 2020). In recent years, several studies have combined iron toxicity measures with other therapeutic approaches to kill tumor cells, that is, introducing other strategies with ferroptosis for more efficient multi-modal carcinoma therapy (Liu et al., 2018; Zheng et al., 2021c). Our results indicated that metallo-organic supramolecular realized ferroptosis sensitization through p53 pathway reactivation and provided a feasible delivery scheme for p53-mediated tumor ferroptosis death. As described above, supramolecular therapeutic agents have been extensively developed in cancer therapy to elevate target specificity and treatment efficacy and, at the same time, reduce the side effects on normal cells.

In conclusion, under the combination of peptide chemistry and nanotechnology, we developed an intracellular-activatable nanoparticle for promoting p53 of the tumor cells and combining ferroptosis and apoptosis. Here, Nano-PMI@CeO_2_ showed the enormous potential of metallo-organic supramolecular in restoring the p53 pathway *in vitro* and *in vivo*, primed the tumor cells to cell apoptosis, and augmented GPX4-related ferroptosis. This is an effective attempt to apply metal-organic supramolecules to sensitize tumor iron death. Taken together, this study not only validated sensitizing ferroptosis *via* reactivation p53 as a clinical translational potential but also more importantly provided a practicable pattern to translate metal-organic supramolecules into a candidate drug for tumor-targeted strategy.

## Data Availability

The original contributions presented in the study are included in the article/Supplementary Material; further inquiries can be directed to the corresponding authors.
