# Supplementary material for: Assembling p53 Activating Peptide With CeO2 Nanoparticle to Construct a Metallo-Organic Supermolecule Toward the Synergistic Ferroptosis of Tumor
Source: Front Bioeng Biotechnol. 2022 Jun 28;10:929536. doi: 10.3389/fbioe.2022.929536 (PMC9273839; doi:10.3389/fbioe.2022.929536)
Supplement: Supplementary file 1 [file DataSheet1.docx]

Supplementary materials

**Assembling p53 activating peptide with CeO_2_ nanoparticle to construct a metallo-organic supermolecule toward the synergistic** **ferroptosis of tumor**

Jingmei Wang^1, †^, Wenguang Yang^2,3, †^, Xinyuan He^4^, Zhang Zhang^5, *^, and Xiaoqiang Zheng^1,2, *^

1. Institute for Stem Cell & Regenerative Medicine, The Second Affiliated Hospital of Xi’an Jiaotong University, Xi’an 710004, China.

2. Department of Medical Oncology, The First Affiliated Hospital of Xi'an Jiaotong University, Xi’an 710061, China.

3. Department of Talent Highland, The First Affiliated Hospital of Xi’an Jiao Tong University, Xi’an 710061, China.

4. Department of Infectious Diseases, The Second Affiliated Hospital of Xi'an Jiaotong University, Xi’an 710004, China.

5. General Surgery Department, Tang Du Hospital, Fourth Military Medical University, 710032 Xi’an, Shaanxi, China

^†^ These authors contributed equally.

^*^ Corresponding authors:

Email: [zhengxiaoqiang@xjtu.edu.cn](mailto:zhengxiaoqiang@xjtu.edu.cn) (X. Zheng)

Email: pwzhangz@fmmu.edu.cn (Z. Zhang)

1. **Supplementary Figures.**


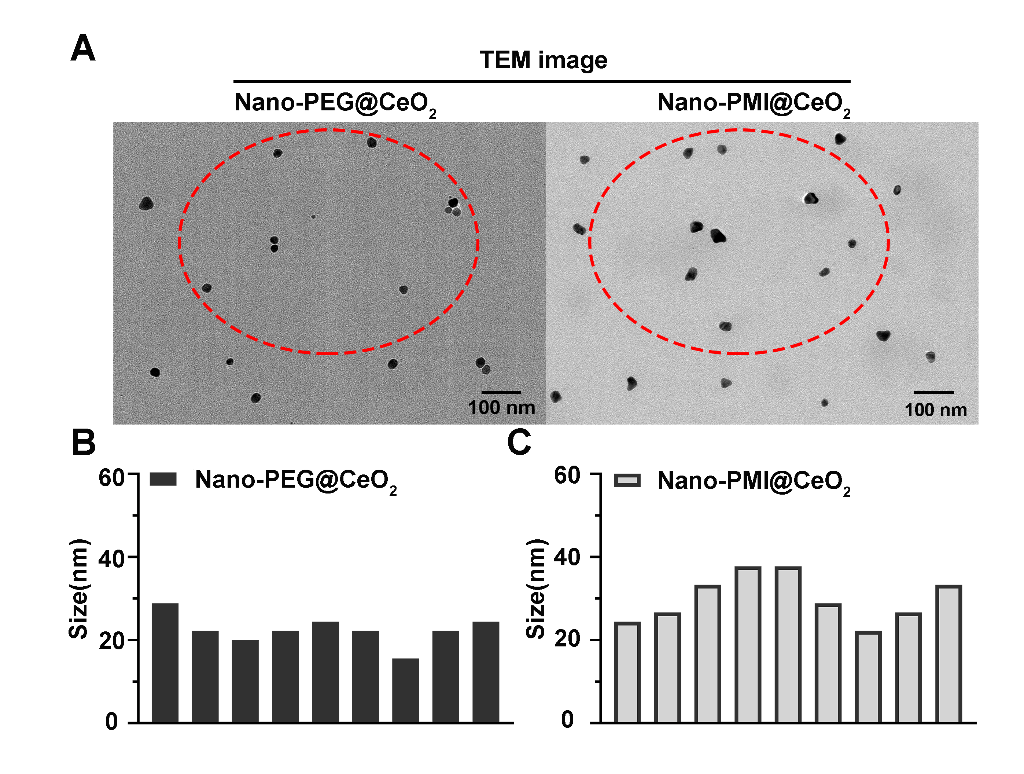


**Figure S1. (A)**TEM images of Nano-PEG@CeO_2_ and Nano-PMI@CeO_2_. **(B&C)** The size distribution of nanoparticles that marked with red circle was shown by the TEM technique.


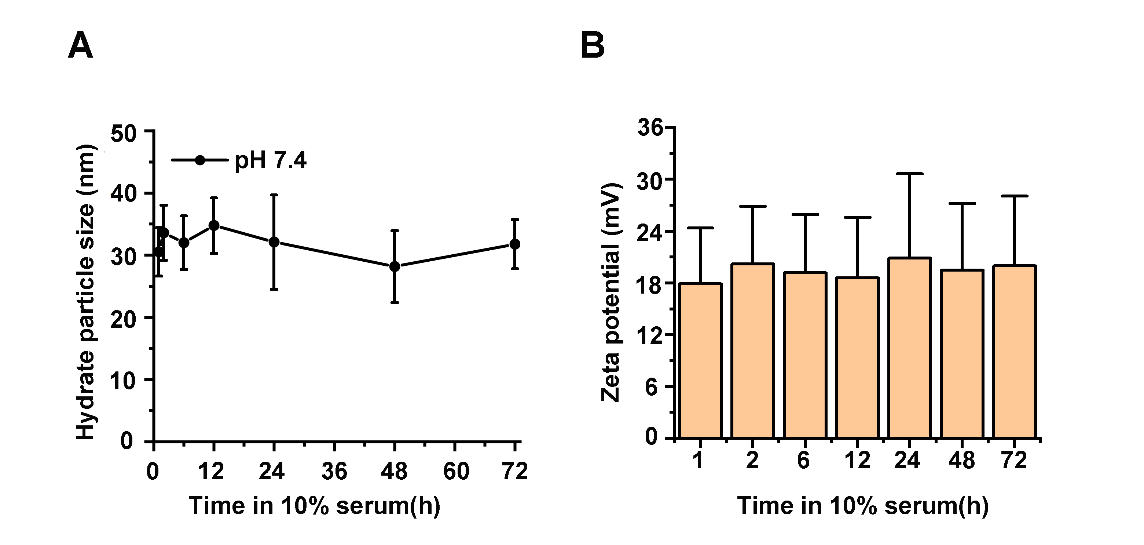


**Figure S2. (A&B)** The stability of Nano-PMI@CeO_2_ in 10% serum solution (pH 7.4) at different time points was measured by DLS.


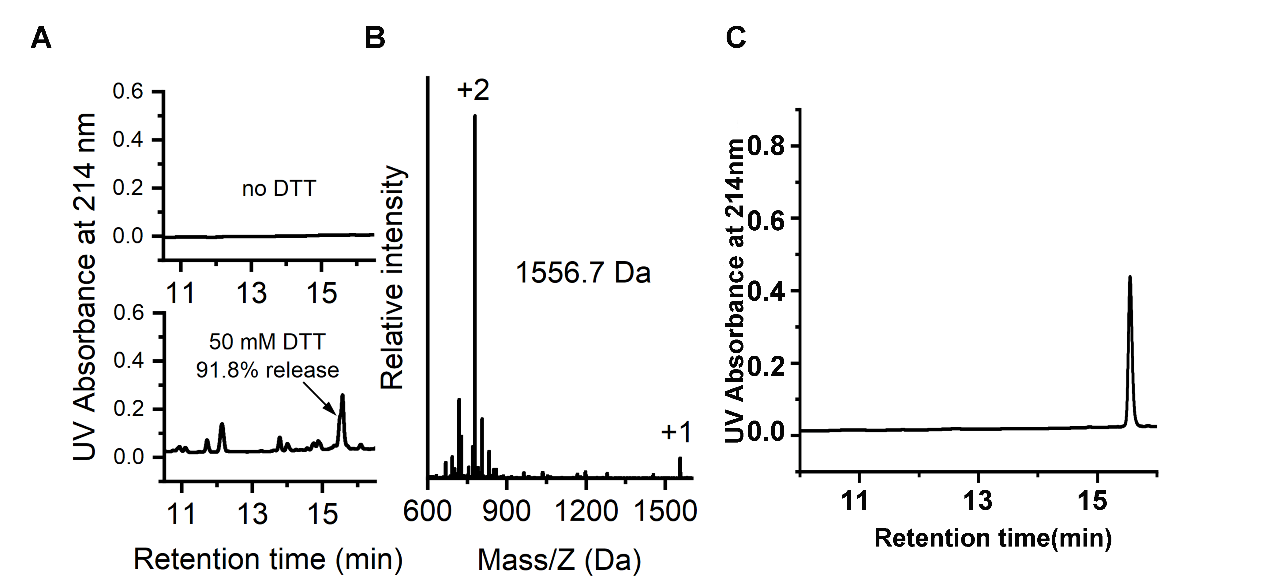


**Figure S3. (A)** The released Nano-PMI@CeO_2_ with 50 mM DTT solution measured via Liquid chromatography. **(B)** PMI peptide relative molecular mass was detected by mass spectrometry. **(C)** HPLC chromatography of PMI polypeptide standard solution (concentration: 0.2 mg/mL).


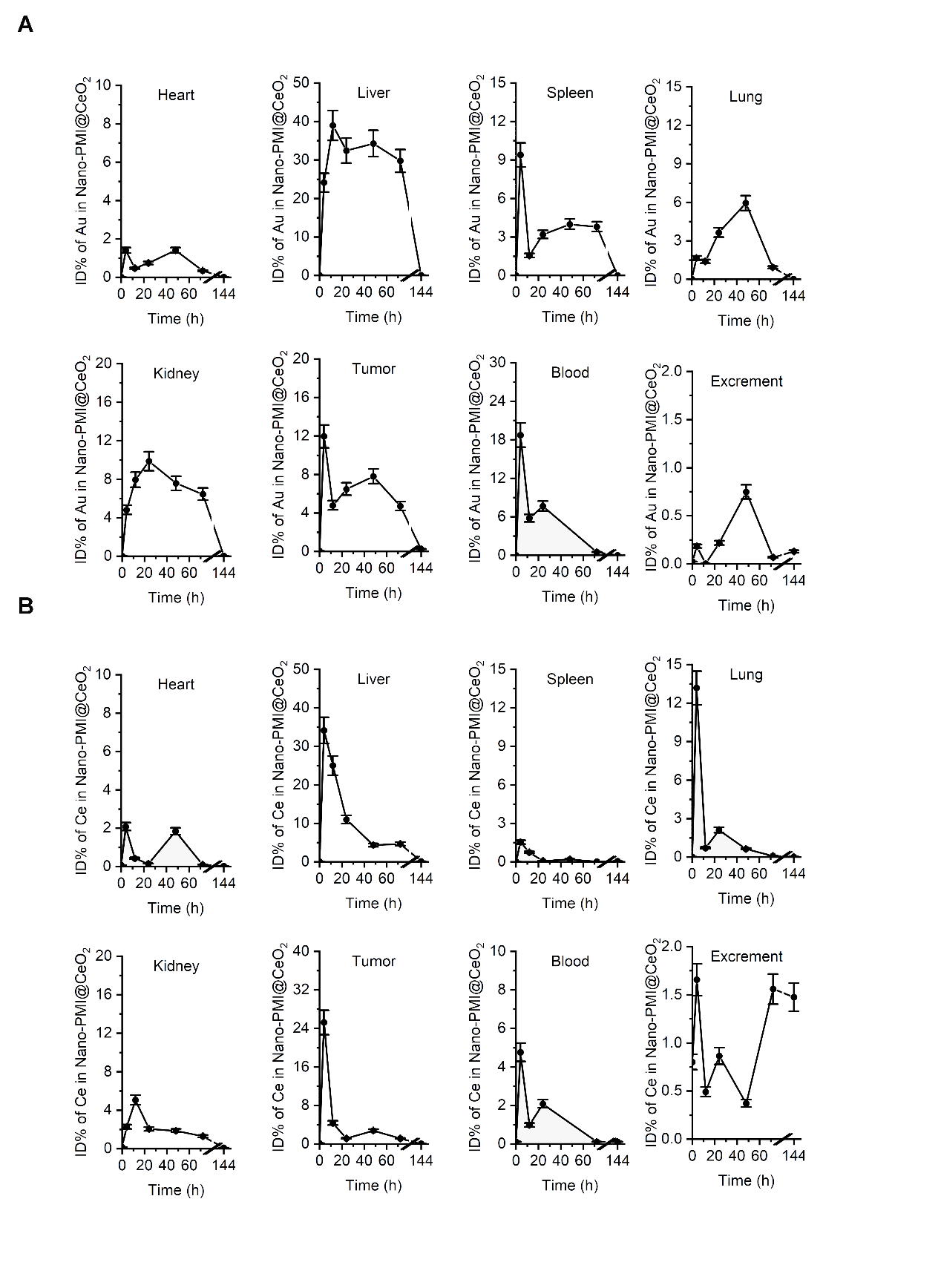


**Figure S4**. (A) Au content of Nano-PMI@CeO_2_ in tumors, organs, and faces originated from LLC-bearing mice by ICP-Mass quantitative analysis. (B) Ce content of Nano-PMI@CeO_2_ in tumors, organs, and faces originated from LLC-bearing mice by ICP-Mass quantitative analysis.


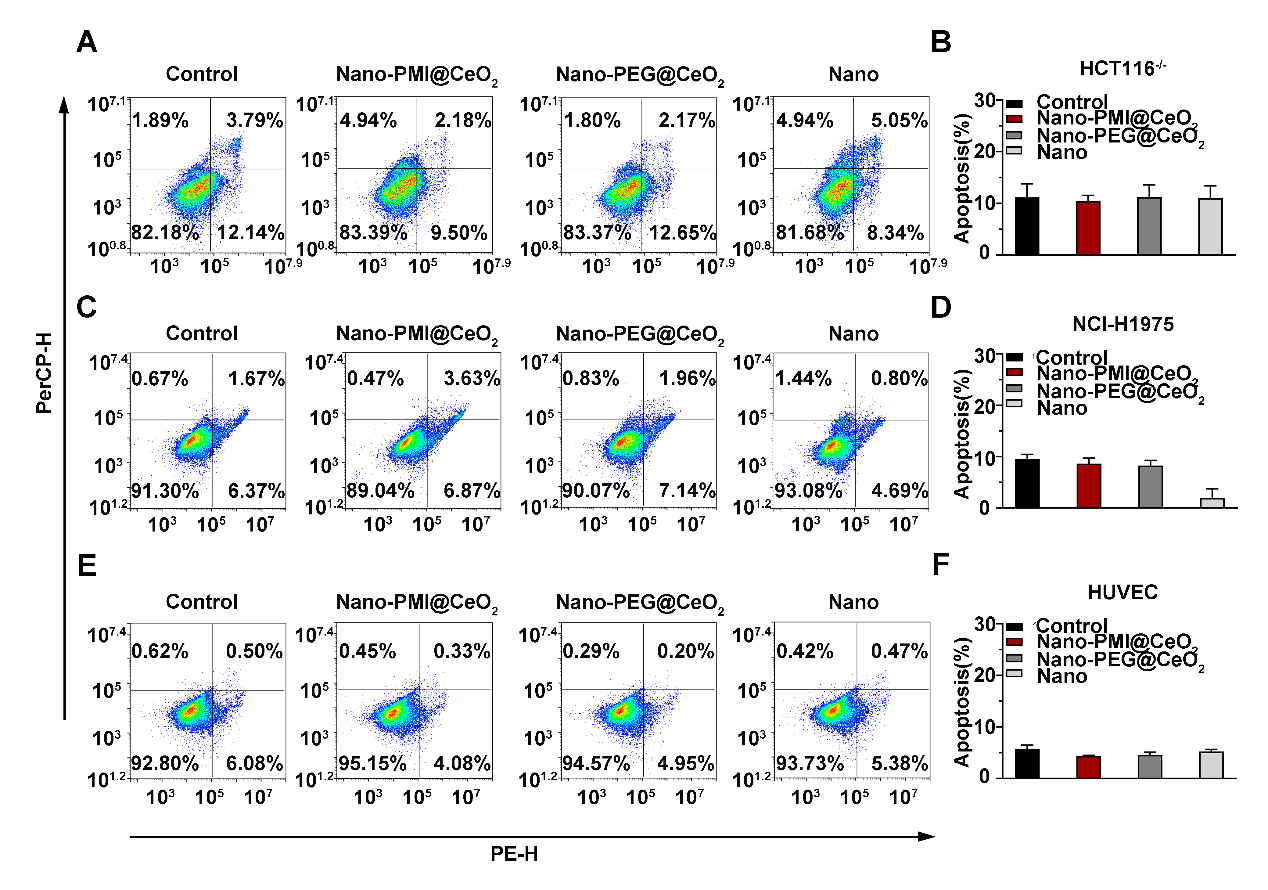


**Figure S5.** (A) Apoptosis effects of these NPs on the HCT116^-/-^ cell line measured by flow cytometric analysis. (B) Apoptosis rate was shown as mean±SE(n=3). (C) Apoptosis effects of these NPs on the NCI-H1975 cell line measured by flow cytometric analysis. (D) Apoptosis rate was shown as mean±SE(n=3). (E) Apoptosis effects of these NPs on the HUVEC cell line measured by flow cytometric analysis. (F) Apoptosis rate was shown as mean±SE(n=3).

‘





**Figure S6.** Protein expression was showed of MDM2 in A549 cell line treatment with 0.02 mg/mL Nano-PMI@CeO_2_, 0.02 mg/mL Nano-PEG@CeO_2_ using western blot.


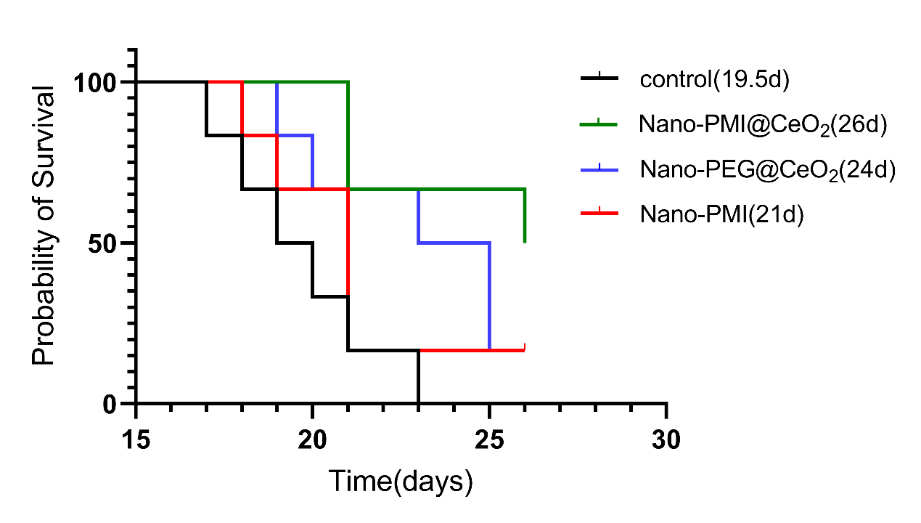


**Figure S7.** Survival image for mice after inoculation of LLC cell line with Control, [Nano-PMI@CeO_2_(2 mg/kg), Nano-PEG@CeO_2_(2 mg/kg)](mailto:Nano-PMI@CeO2(2.5mg/kg),%20Nano-PEG@CeO2(2.5mg/kg)) and Nano-PMI(2 mg/kg) treatments (n=6).


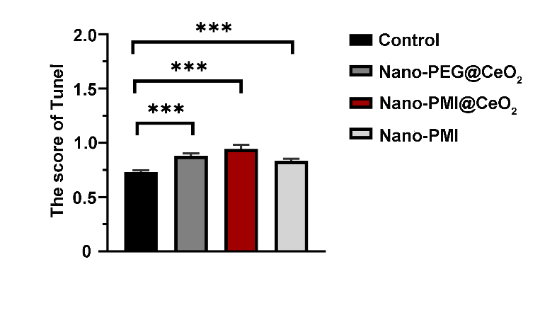


**Figure S8**. Relative quantitative analysis of Tunel staining. *P* values were calculated by *t*-test(***, *p*＜0.001).

**2. Supplementary Tables.**

**Table S1**. The ratio of elements Au and Ce in the nanoparticles

| **Element** | **Au** | **Ce** |
| --- | --- | --- |
| Supernatant concentration | 4.361 mg/L | 0.154 mg/L |
| Supernatant ratio | 2.2 % | 0.4 % |
| Ratio in nanoparticles | 97.8 % | 99.6 % |
